# Clinical Psoriasiform Dermatitis Following Dupilumab Use for Autoeczematization Secondary to Chronic Stasis Dermatitis

**DOI:** 10.7759/cureus.7831

**Published:** 2020-04-25

**Authors:** Kory P Schrom, Alison Kobs, Susan Nedorost

**Affiliations:** 1 Dermatology, University Hospitals Cleveland Medical Center, Cleveland, USA

**Keywords:** autoeczematization, dupilumab, psoriasiform, th2, th1

## Abstract

T helper 2 (Th2) and T helper 1 (Th1) mediated immune processes lie on a spectrum. Autoeczematization secondary to chronic stasis dermatitis may fall on the Th2 side of the spectrum due to skin stretch and chronic barrier dysfunction, supporting a primary Th2 response to self-antigen. In our patient, we posited that dupilumab would benefit autoeczematization secondary to chronic stasis dermatitis given its efficacy in atopic dermatitis, a Th2-mediated immune process. We report a case of clinical psoriasiform dermatitis, suggesting a shift toward a Th1-mediated immune process developing during dupilumab treatment for autoeczematization secondary to chronic stasis dermatitis.

## Introduction

Patients with chronic venous stasis dermatitis are susceptible to developing autoeczematization, with an associated 37% lifetime prevalence [1,2]. The cause of autoeczematization is likely due to chronic skin stretch, skin irritation, and chronic barrier dysfunction supporting autosensitization to self-antigens [3]. We hypothesize that these protein self-antigens promote a T helper 2 (Th2) mediated immune response, which is a phenomenon seen with other protein allergens in disease states such as atopic dermatitis [4]. Therefore, a “skewing” of the immune response to a predominately Th2-mediated immune process in a patient with autoeczematization secondary to chronic stasis dermatitis is theoretically possible [5].

Mediators of a Th2-mediated immune process include interleukin (IL)-4 and IL-13, which are targeted by the monoclonal antibody, dupilumab [6,7]. Given the mechanism of action for dupilumab and the hypothesized pathogenesis of autoeczematization, it is possible that dupilumab could be effective in treating autoeczematization. We report here the first case of clinically developed psoriasiform dermatitis during dupilumab treatment for autoeczematization secondary to chronic stasis dermatitis.

## Case presentation

An 80-year-old male with autoeczematization secondary to chronic stasis dermatitis developed clinical psoriasiform dermatitis after treatment with dupilumab. He initially presented with generalized pruritic papules and eczematous patches on the trunk, arms, and lower extremities in the setting of previously diagnosed chronic lower extremity swelling and venous stasis dermatitis. He rated his itch a 10 (i.e. worst possible), and a biopsy of the right upper back revealed subacute spongiosis with frequent eosinophils. Direct immunofluorescence was negative for immunoglobulin (Ig) G, IgA, IgM, complement 3, and fibrinogen. Indirect immunofluorescence was negative for BP230/180 autoantibodies. Extensive patch testing was performed and was non-contributory as there was no improvement with topical and systemic avoidance of items producing positive patch test results. A diagnosis of autoeczematization secondary to chronic stasis dermatitis was made. Unna boots were initiated followed by daily compression stocking use and triamcinolone 0.1% ointment as needed. With therapy, his skin improved and his itch was rated a 2. He was instructed to follow-up as needed.

He returned several months later following a prolonged car ride with a similar clinical picture of generalized pruritic papules and eczematous patches on the trunk, arms, and lower extremities. He also had pitting edema and rated his itch a 9. Despite adequate compression with optimal patient compliance, triamcinolone as needed, and several courses of prednisone, his condition was uncontrolled; therefore, our patient asked if dupilumab might help. We reasoned that chronic stasis dermatitis might provoke a Th2-skewed immune response to self-antigen in skin experiencing stretch and that mechanistically dupilumab may help.

**Figure 1:**
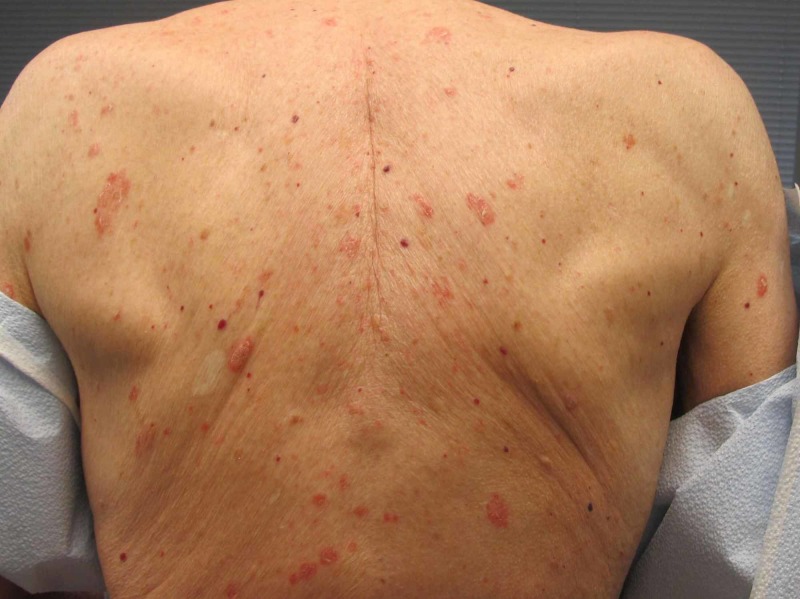
Scaling erythematous psoriasiform plaques on the upper back after 10 weeks of dupilumab therapy.

The patient was treated with dupilumab 300 mg every two weeks subcutaneously with an initial loading dose of 600 mg. An “Investigative New Drug” designation and Institutional Review Board approval were obtained for this off-label use. After 10 weeks, itch decreased to 3 out of 10 and he had significantly reduced lower extremity edema; however, the morphology transitioned from papules and patches to scaling erythematous plaques more consistent with clinical psoriasiform dermatitis (Figure 1).

Dupilumab was discontinued, and a repeat biopsy of the right upper posterior arm was performed at the time of discontinuation. The biopsy demonstrated non-specific histology with orthokeratosis overlying parakeratosis, very mild spongiosis, lymphocyte exocytosis, and a mild superficial perivascular lymphocytic infiltrate. Per the dermatopathologist, early psoriasis could not be excluded, and the histology needed to be correlated with the clinical picture. The patient was subsequently started on narrowband ultraviolet B phototherapy and responded well.

## Discussion

Clinical psoriasiform dermatitis developed in our patient after treatment with dupilumab for autoeczematization secondary to chronic stasis dermatitis. Similar events have been reported in patients with atopic dermatitis who developed psoriasiform dermatitis after treatment with dupilumab, but this is the first case related to autoeczematization [8-12].

We treated our patient with dupilumab because we hypothesized that autoeczematization is driven by a Th2-mediated immune process involving the cytokines IL-4 and IL-13 whose functions are inhibited by dupilumab [6,13]. Skin stretch in chronic stasis dermatitis results in chronic barrier dysfunction, which supports an altered epidermal microenvironment that is primed for induction of a Th2-immune response, as described by Ellenbogen et al. [7,14]. In the setting of this primed microenvironment, sensitization to autologous skin proteins occurs and subsequently promotes autoeczematization [3,14]. Thus, dupilumab was considered a reasonable treatment for our patient. Although his itch improved after 10 weeks of therapy, his clinical morphology became psoriasis-like.

Although our patient’s biopsy was non-specific for psoriasis, biopsies related to dupilumab-induced psoriasiform dermatitis may not always demonstrate clear psoriasiform changes even when the lesions clinically appear psoriasiform. Fowler et al. reported a case that appeared as psoriasiform dermatitis clinically but histologically as acute spongiotic dermatitis [11]. It is also possible that clinical presentation may not be psoriasiform in appearance; but histologically, psoriasiform changes can be present [15]. Providers, therefore, must review the entire clinical picture when diagnosing and treating this phenomenon.

Psoriasis and atopic dermatitis have been described as sitting on a spectrum between T helper 1 (Th1) cell immunity and Th2 cell immunity. A shift toward Th1 immunity drives psoriasis, and a shift toward Th2 immunity drives atopic dermatitis [13]. We postulate that dupilumab shifted our patient along this spectrum toward a more Th1-mediated process through its inhibition of IL-4 and IL-13 signaling [6]. IL-4 has been shown to enhance Th2 responses and acts as a negative regulator of Th1 immune responses related to psoriasis through direct action on T cells, keratinocytes, and dendritic cells [10,16].

Interestingly, a reverse phenomenon has been reported where patients with psoriasis developed eczematous eruptions after treatment with TNFα (tumor necrosis factor-α) inhibitors and the anti-IL-17 monoclonal antibody, secukinumab [17,18]. These shifts in phenotypic presentation between atopic dermatitis and psoriasis are not likely to occur in every patient, but previous research demonstrates that some patients may be at an increased risk due to genetic susceptibilities to both atopic dermatitis and psoriasis [19,20].

## Conclusions

Chronic stasis dermatitis causes skin stretch, resulting in chronic barrier dysfunction. This dysfunction subsequently supports a Th2-mediated immune response to autologous skin proteins and the development of autoeczematization. Dupilumab is effective in treating atopic dermatitis, a Th2-mediated process, but it may skew the immune response to a Th1-skewed response in less Th2-dominant processes such as autoeczematization secondary to chronic stasis dermatitis. Further research is required to better understand this phenomenon.
